# Efficacy and safety of abemaciclib alone and with PI3K/mTOR inhibitor LY3023414 or galunisertib versus chemotherapy in previously treated metastatic pancreatic adenocarcinoma: A randomized controlled trial

**DOI:** 10.1002/cam4.6621

**Published:** 2023-10-16

**Authors:** E. Gabriela Chiorean, Vincent Picozzi, Chung‐Pin Li, Marc Peeters, Joan Maurel, Jaswinder Singh, Talia Golan, Jean‐Frédéric Blanc, Sonya C. Chapman, Anwar M. Hussain, Erica L. Johnston, Howard S. Hochster

**Affiliations:** ^1^ University of Washington School of Medicine Seattle Washington USA; ^2^ Fred Hutchinson Cancer Center Seattle Washington USA; ^3^ Virginia Mason Hospital and Medical Center Seattle Washington USA; ^4^ Division of Clinical Skills Training, Department of Medical Education Taipei Veterans General Hospital Taipei Taiwan; ^5^ Division of Gastroenterology and Hepatology, Department of Medicine Taipei Veterans General Hospital Taipei Taiwan; ^6^ Therapeutic and Research Center of Pancreatic Cancer Taipei Veterans General Hospital Taipei Taiwan; ^7^ School of Medicine, College of Medicine National Yang Ming Chiao Tung University Taipei Taiwan; ^8^ Department of Oncology Antwerp University Hospital Antwerp Belgium; ^9^ Department of Oncology, Faculty of Medicine and Health Sciences University of Antwerp Antwerp Belgium; ^10^ Medical Oncology Department, Hospital Clinic of Barcelona, Translational Genomics and Targeted Therapeutics in Solid Tumors Group, IDIBAPS University of Barcelona Barcelona Spain; ^11^ Sarah Cannon Cancer Institute at Research Medical Center Kansas City Missouri USA; ^12^ Oncology Institute, Sheba M9edical Center at Tel‐Hashomer Tel Aviv University Tel Aviv Israel; ^13^ Service d'Hépato‐Gastroentérologie et d'Oncologie Digestive Groupe Hospitalier Haut‐Lévêque CHU Bordeaux Pessac France; ^14^ Eli Lilly and Company Indianapolis Indiana USA; ^15^ Rutgers Cancer Institute of New Jersey New Brunswick New Jersey USA

**Keywords:** Abemaciclib, metastatic pancreatic cancer, PI3K/mTOR, TGFβ, second‐line therapy, third‐line therapy

## Abstract

**Background:**

Pancreatic ductal adenocarcinomas (PDAC) are characterized by frequent cell cycle pathways aberrations. This study evaluated safety and efficacy of abemaciclib, a cyclin‐dependent kinase 4 and 6 inhibitor, as monotherapy or in combination with PI3K/mTOR dual inhibitor LY3023414 or TGFβ inhibitor galunisertib versus standard of care (SOC) chemotherapy in patients with pretreated metastatic PDAC.

**Methods:**

This Phase 2 open‐label study enrolled patients with metastatic PDAC who progressed after 1–2 prior therapies. Patients were enrolled in a safety lead‐in (abemaciclib plus galunisertib) followed by a 2‐stage randomized design. Stage 1 randomization was planned 1:1:1:1 for abemaciclib, abemaciclib plus LY3023414, abemaciclib plus galunisertib, or SOC gemcitabine or capecitabine. Advancing to Stage 2 required a disease control rate (DCR) difference ≥0 in abemaciclib‐containing arms versus SOC. Primary objectives for Stages 1 and 2 were DCR and progression‐free survival (PFS), respectively. Secondary objectives included response rate, overall survival, safety, and pharmacokinetics.

**Results:**

One hundred and six patients were enrolled. Abemaciclib plus galunisertib did not advance to Stage 1 for reasons unrelated to safety or efficacy. Stage 1 DCR was 15.2% with abemaciclib monotherapy, 12.1% with abemaciclib plus LY3023414, and 36.4% with SOC. Median PFS was 1.7 months (95% CI: 1.4–1.8), 1.8 months (95% CI: 1.3–1.9), and 3.3 months (95% CI: 1.1–5.7), respectively. No arms advanced to Stage 2. No new safety signals were identified.

**Conclusion:**

In patients with pretreated metastatic PDAC, abemaciclib‐based therapy did not improve DCRs or PFS compared with SOC chemotherapy. No treatment arms advanced to Stage 2. Abemaciclib remains investigational in patients with PDAC.

## Graphical abstract

Pancreatic ductal adenocarcinomas (PDAC) are characterized by frequent cell cycle pathways aberrations. This phase 2 randomized study for refractory metastatic PDAC patients showed that the CDK4/6 inhibitor abemaciclib, alone or in combination with the PI3K/mTOR LY3023414, does not improve disease control or progression‐free survival compared to standard chemotherapy.

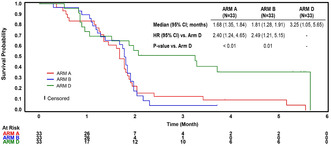

## 1 INTRODUCTION

Pancreatic ductal adenocarcinoma (PDAC) is highly lethal and the fourth leading cause of cancer deaths worldwide, with a 5‐year survival of only 10%.^1^, ^2^ The majority of PDAC patients are initially diagnosed with locally advanced or metastatic disease.^3^ Pancreatic ductal adenocarcinoma is predominantly a chemotherapy‐resistant disease, partly due to multiple genetic aberrations and an immunosuppressive environment, among other factors. Overall survival for metastatic disease ranges between 8.5 and 14 months with first‐line multiagent chemotherapy,^4^, ^5^, ^6^, ^7^ 6–8 months with second‐line therapies,^8^, ^9^, ^10^ and 3–5 months with third‐line therapies.^10^, ^11^, ^12^ Despite therapeutic advances, the optimal second‐line strategy remains unknown, and currently, no standard third‐line treatment exists.^13^ Thus, novel targeted therapies geared towards the most common molecular alterations are needed.^14^

*KRAS* mutations occur in nearly 95% of PDACs early in tumorigenesis.^15^ Clinical trials exploring the efficacy of agents targeting the *RAS* pathway have been largely unsuccessful, with the exception of KRAS G12C inhibitors, mainly due to downstream compensatory mechanisms.^16^ Additionally, G1/S cell cycle transition is commonly activated due to loss, mutation, or epigenetic silencing of the cyclin‐dependent kinase (CDK) inhibitor 2A (CDKN2A) or activating mutations or overexpression of CDK4, CDK6, or D‐type cyclins.^15^, ^16^, ^17^ *CDKN2A* encodes the tumor suppressor protein p16INK4A, an endogenous inhibitor of CDK4 and CDK6, which results in reduced Rb phosphorylation and G1 cell cycle arrest. Moreover, many oncogenes, including *KRAS*, lead to G1/S pathway activation.^15^, ^16^, ^17^

Preclinical data with the CDK4/6 inhibitors, palbociclib and abemaciclib, demonstrated variable single‐agent efficacy in PDAC models.^18^, ^19^ While modest activity was observed, inhibition of CDK4/6 led to adaptive responses with increased mammalian target of rapamycin (mTOR) expression promoting resistance to palbociclib, remedied by dual inhibition of CDK4/6 and mTOR.^20^ In addition, preclinical pancreatic cancer models demonstrated increased epithelial‐to‐mesenchymal transition and cell invasion driven by transforming growth factor beta (TGFβ).^21^ Inhibition of the type‐1 TGFβ receptor (TGFβ‐R1) kinase in combination with CDK4/6 decreased cell invasion and inhibited tumor growth in cell line models. CDK4/6 inhibitors in combination with phosphatidylinositol 3‐kinase (PI3K)/mTOR or TGFβ receptor type I (TGFβ‐RI) inhibitors have confirmed efficacy in preclinical pancreatic cancer models.^21^, ^22^

Abemaciclib is a potent, selective small‐molecule CDK4/6 inhibitor and is FDA‐approved as monotherapy and in combination with endocrine therapy for hormone receptor positive (HR+) and human epidermal growth factor receptor 2 negative (HER2‐) advanced breast cancer,^23^, ^24^, ^25^ and for the adjuvant treatment of HR+, HER2‐, node‐positive, early breast cancer at high risk of recurrence.^26^, ^27^

Given the relevance of the CDK4/6 pathway in PDAC, the preclinical activity of CDK4/6 inhibition in PDAC models, including in *KRAS* mutant pancreatic cancer cell lines,^19^ acceptable safety and tolerability, and the unmet medical need for refractory PDAC, this study aimed to explore the safety, efficacy, and pharmacokinetics (PK) of abemaciclib monotherapy and in combination with agents targeting PI3K/mTOR or TGFβ pathways compared to standard of care (SOC) chemotherapy in second‐ or third‐line treatment of patients with metastatic PDAC.

## 2 METHODS

### 2.1 Study design

Study I3Y‐MC‐JPCJ was an international, multicenter, adaptive, randomized, open‐label, Phase 2 study in patients with metastatic PDAC who had disease progression after 1 or 2 prior therapies. The study aimed to evaluate the safety and efficacy of abemaciclib monotherapy or in combination with targeted agents versus standard chemotherapy of physician's choice, using a 2‐stage design (Figure S1). Prior to initiating the 2‐stage randomization portion, a safety lead‐in was planned to assess the initial safety of abemaciclib plus galunisertib in up to 12 patients. However, testing of this combination was stopped by the sponsor for reasons unrelated to safety. Following an amendment, no additional patients were enrolled in the safety lead‐in, and the 2‐stage randomization phase was initiated without the abemaciclib plus galunisertib arm.

Dose selection for abemaciclib as monotherapy and in combination with LY3023414 or with galunisertib, as well as doses for LY3023414 and galunisertib were based on previously reported Phase 1 studies.^28^, ^29^, ^30^, ^31^ Stage 1 is planned to randomize approximately 25 patients each 1:1:1 to receive either abemaciclib monotherapy (Arm A), abemaciclib plus LY3023414 (Arm B), or physician's choice SOC with either gemcitabine or capecitabine (Arm D). Following completion of Stage 1, any treatment arms that demonstrated a higher disease control rate (DCR = complete response [CR] + partial response [PR] + stable disease [SD]) than was achieved in the SOC arm were to advance to Stage 2 and randomize an additional 50 patients to each respective arm. Enrollment in non‐advancing arms would be discontinued.

### 2.2 Patients

Eligible patients were ≥18 years of age with metastatic PDAC and disease progression following 1 or 2 prior lines of therapy. Patients were required to have measurable disease as defined by RECIST v1.1, an Eastern Cooperative Oncology Group (ECOG) performance status (PS) of 0 or 1, adequate organ function, and be considered appropriate candidates for single‐agent chemotherapy with capecitabine or gemcitabine. Patients with insulin‐dependent diabetes mellitus, symptomatic central nervous system metastases, or those previously treated with CDK4/6, PI3K, and/or mTOR inhibitors were not eligible.

The study protocol was approved by the appropriate institutional review boards and ethics committees and conducted in accordance with the Good Clinical Practice of the Declaration of Helsinki. All patients provided written informed consent. Patients were enrolled at 32 sites in 8 countries.

### 2.3 Randomization and treatment

An interactive web response system assigned treatment. Investigational treatments were administered orally with or without food on a 28‐day cycle, unless otherwise noted. Standard chemotherapy was administered according to prescribing label recommendations. Treatment was continued until progressive disease (PD), unacceptable toxicity, death, or withdrawal from the study.

In the safety lead‐in, patients received abemaciclib continuously (150 mg twice daily [BID]) plus galunisertib (150 mg BID) for 14 days followed by a 14‐day rest period. In Stage 1, patients were randomized 1:1:1 to receive abemaciclib monotherapy (200 mg twice daily), abemaciclib (150 mg BID) plus LY3023414 (150 mg BID), or SOC chemotherapy of the physician's choice with gemcitabine (1000 mg/m^2^ on Days 1, 8, 15, and 22 [Cycle 1 only], followed by Days 1, 8, and 15) or capecitabine (1250 mg/m^2^ BID within 30 min after a meal [morning and evening] on Days 1–14 on 21‐day cycles). The same dosing schedules were to be used in Stage 2 for any advancing treatment arm(s). Stratification was based on the number of prior systemic therapies.

Dose modifications of investigational agents were allowed for treatment‐related toxicities and followed protocol guidance. Standard chemotherapy dose modifications followed on‐label recommendations.

Patients received full supportive care during the study per institutional guidelines. The use of granulocyte‐colony stimulating factors and erythropoietin was permitted in accordance with American Society of Clinical Oncology/American Society of Hematology guidelines.^32^, ^33^

### 2.4 Safety and efficacy assessments

Adverse events (AEs) were collected and graded using the National Cancer Institute Common Terminology Criteria for Adverse Events Version 4.0.^34^ Tumor assessments were conducted using computed tomography or magnetic resonance imaging scans according to RECIST v1.1 at screening (within 28 days prior to randomization) and approximately every 8 weeks thereafter.^35^

### 2.5 Pharmacokinetics

For patients receiving abemaciclib, PK samples were collected on Cycle 1 Day 1 approximately 2 h after dosing and pre‐dosed on Day 1 of Cycles 2, 3, and 4. During the safety lead‐in, PK samples were collected pre‐dose on Cycle 1 Days 1 and 14, and at 0.5‐, 1‐, 2‐, 4‐, 6‐, and 8‐h post‐dose. Pharmacokinetics samples were analyzed for galunisertib, abemaciclib, and its metabolites (LSN2839567 [M2] and LSN3106726 [M20]) (Q^2^ Solutions) and LY3023414 (Covance Laboratories Inc.) using validated liquid chromatography with tandem mass spectrometric (LC/MS/MS) methods.

For the safety lead‐in, non‐compartmental analysis methods were used to compute standard PK parameters of abemaciclib, M2, M20, and galunisertib. For Stage 1, average concentrations of abemaciclib, M2, M20, and LY3023414 were reported at each planned PK sampling time.

### 2.6 Endpoints

Stage 1 primary endpoint was disease control rate (DCR) defined as the proportion of patients with a best tumor response of CR, PR, or SD (DCR = CR + PR + SD) in the intent‐to‐treat population. Responses and SD did not require confirmation. Secondary endpoints included ORR (CR + PR), pharmacokinetics (PK) of abemaciclib, its metabolites and LY3023414, and safety.

Stage 2 primary endpoint of progression‐free survival (PFS) was defined as the time from randomization until progression or death from any cause. Secondary endpoints included DCR, clinical benefit rate (CR + PR + SD ≥6 months), ORR, duration of response (time from response until progression or death), and overall survival (OS).

### 2.7 Statistical analyses

The study had a two‐stage design. During Stage 1, approximately 25 patients per arm were planned to provide a preliminary assessment of DCR and safety. The DCR of abemaciclib (Arm A) and abemaciclib plus LY3023414 (Arm B) were compared to SOC chemotherapy (Arm D). The null hypothesis assumed the DCR with SOC or abemaciclib treatment to be 50%. The probability of stopping at the end of Stage 1 was 11% if the DCR with abemaciclib treatment was 65% (i.e., DCR difference of abemaciclib vs. SOC was +15%). Conversely, the probability of stopping at the end of Stage 1 was 72% if the DCR with abemaciclib was 40% (i.e., DCR Difference of abemaciclib vs. SOC was −10%). At the end of Stage 1, an additional 50 patients were planned to enroll in each treatment arm with a DCR at least as good as SOC (i.e., DCR difference of abemaciclib vs. SOC ≥0), totaling approximately 75 patients in each arm. All efficacy analyses were performed on the intent‐to‐treat (ITT) population (all randomized patients). Stage 1 analysis was performed approximately 16 weeks after the last patient entered treatment.

All tumor assessments were used to determine DCR and ORR. Each of these rates, point estimates, and confidence intervals (CIs) (using the normal approximation to the binomial) were calculated by the treatment arm. Kaplan–Meier (KM) method was used to estimate the PFS and OS for each treatment arm.^36^ Comparison between abemaciclib‐containing arms and SOC were done using the log‐rank test stratified by randomization strata. Stratified Cox proportional hazard model was used to estimate the HR and its corresponding 95% CI.

The safety population included all patients who received any study treatment. Safety data were summarized by treatment arms. The PK population included patients who had ≥1 evaluable PK sample.

## 3 RESULTS

### 3.1 Patients

**FIGURE 1:**
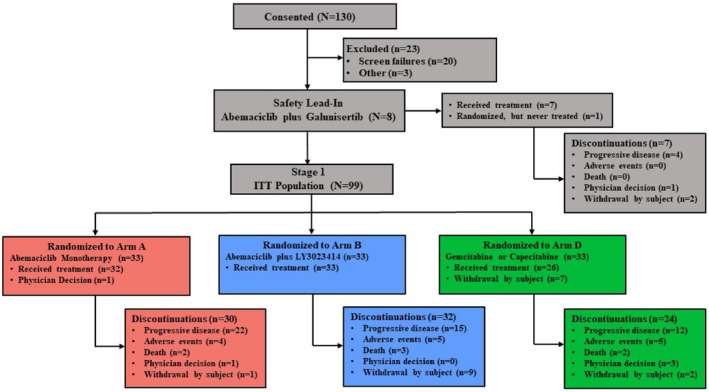
Summary of patient disposition.

From January 2017 to December 2017, 7 patients were treated with abemaciclib plus galunisertib (safety lead‐in), and 99 patients were randomized to receive abemaciclib (Arm A: *n* = 33), abemaciclib plus LY3023414 (Arm B: *n* = 33), or SOC chemotherapy (Arm D: *n* = 33) (Figure 1). Patients already in screening at the time of enrollment closure were allowed to continue and enroll if eligible; thus, more than 25 planned patients were enrolled in each arm. A total of 91 patients received study treatment (Arm A: *n* = 32, Arm B: *n* = 33, Arm D: *n* = 26); 8 patients were randomized but did not receive treatment, of which most were due to patient withdrawal of consent from the SOC arm after randomization (Arm D: *n* = 7) and 1 physician decision (Arm A) (Figure 1). The data cutoff date was April 20th, 2018. Baseline and disease characteristics were well balanced (Table 1). Most patients (84.9%) received prior gemcitabine‐based therapy, and 60.4% received prior fluoropyrimidine‐based therapy. Forty‐eight patients (45.3%) received 1, and 58 (54.7%) had two previous lines of systemic therapy for metastatic disease.

**TABLE 1:** Demographics and baseline characteristics (safety lead‐in and ITT population).

	Abemaciclib + Galunisertib (*N* = 8)^a^	Abemaciclib (*N* = 33)	Abemaciclib + LY3023414 (*N* = 33)	Gemcitabine or capecitabine (*N* = 33)
Sex, *n* (%)				
Female	4 (57.1)	18 (54.5)	16 (48.5)	19 (57.6)
Male	3 (42.9)	15 (45.5)	17 (51.5)	14 (42.4)
Age, (years)				
Median (range)	62 (57–77)	61 (47–79)	61 (44–80)	67 (39–85)
Race, *n* (%)				
White	7 (100)	26 (78.8)	26 (78.8)	25 (75.8)
Asian	0	7 (21.2)	5 (15.2)	4 (12.1)
Black or African American	0	0 (0.0)	1 (3.0)	3 (9.1)
Missing	0	0 (0.0)	1 (3.0)	1 (3.0)
Region, *n* (%)^b^				
Europe	0	15 (45.5)	14 (42.4)	14 (42.4)
Asia	0	9 (27.3)	8 (24.2)	8 (24.2)
North America	7 (100)	8 (24.2)	10 (30.3)	8 (24.2)
Australia	0	1 (3.0)	1 (3.0)	3 (9.1)
ECOG PS, *n* (%)^b^				
0	6 (85.7)	15 (45.5)	11 (33.3)	8 (24.2)
1	1 (14.3)	15 (45.5)	18 (54.5)	15 (45.5)
Missing	0	3 (9.1)	4 (12.1)	10 (30.3)
Stage at diagnosis, *n* (%)^b^ ^,^ ^c^				
Stage IB	0	0	1 (3.0)	0
Stage IIA	1 (14.3)	2 (6.1)	1 (3.0)	2 (6.1)
Stage IIB	0	8 (24.2)	10 (30.3)	6 (18.2)
Stage III	0	6 (18.2)	4 (12.1)	4 (12.1)
Stage IV	5 (71.4)	15 (45.5)	17 (51.5)	18 (54.5)
Prior lines of systemic therapy for metastatic disease, *n* (%)^b^				
1	2 (28.6)	15 (45.5)	16 (48.5)	15 (45.4)
2	5 (71.4)	18 (54.5)	17 (51.5)	18 (54.5)
Prior systemic treatments, *n* (%)^d^				
Gemcitabine‐based therapy	6 (85.7)	28 (84.8)	28 (84.8)	28 (84.8)
Fluoropyrimidine‐based therapy	3 (42.9)	17 (51.5)	22 (66.7)	22 (66.7)

Abbreviations: ECOG PS, Eastern Cooperative Oncology Group performance status; *N*, number of patients in arm; *n*, number of patients within category.

^a^ Baseline characteristics are missing for 1 patient.

^b^ Some categories do not add up to 100% due to rounding.

^c^ 6 patients (1 in abemaciclib plus galunisertib, 2 in abemaciclib monotherapy arm, and 3 in SOC arm) had missing stage at diagnosis.

^d^ Categories do not add up to 100% as nearly half of the patients previously received both gemcitabine‐ and fluoropyrimidine‐based therapies.

### 3.2 Treatment

Median duration of abemaciclib treatment was 7.9 weeks (galunisertib safety lead‐in), 6.7 weeks (Arm A), 4.1 weeks (Arm B), 10.6, and 5.1 weeks with gemcitabine and capecitabine (Arm D), respectively. At the data cutoff, a total of 86 patients (86.9%) had discontinued study treatment. Reasons for treatment discontinuation are summarized in Figure 1.

### 3.3 Efficacy

Sixty‐one of 99 patients enrolled (61.6%) were evaluable for response, and 38 patients (38.4%) did not have a post‐baseline scan and were considered non‐evaluable. Of these non‐evaluable patients, 8 (21.1%) were randomized but never treated, 21 (55.3%) died before imaging assessment, and 9 (23.7%) withdrew consent or refused follow‐up prior to the first disease response assessment.

In the ITT population (*N* = 99), the DCR with abemaciclib, abemaciclib plus LY3023414, and SOC were 15.2%, 12.1%, and 36.4%, respectively, favoring SOC chemotherapy (Table 2). Patients receiving abemaciclib or SOC and pretreated with 1 prior systemic therapy (*n* = 15 in both Arms A and D; *n* = 16 in Arm B) for metastatic disease had a better DCR compared to patients who received 2 prior systemic therapies (*n* = 18 in both Arms A and D; *n* = 17 in Arm B) (Arm A: 20.0% vs. 11.1%; Arm B: 6.3% vs. 17.6%; Arm D: 46.7% vs. 27.8%, respectively). Given no improvement in DCR with abemaciclib treatment compared to SOC, Stage 1 futility criteria were met. No treatment arms advanced to Stage 2. Available data were used to analyze all endpoints in the study.

**TABLE 2:** Summary of best overall response (ITT population).

Best overall response	Abemaciclib (*N* = 33)	Abemaciclib + LY3023414 (*N* = 33)	Gemcitabine or capecitabine (*N* = 33)
Disease control rate (CR/PR/SD), *n* (%)	5 (15.2)	4 (12.1)	12 (36.4)
Objective response rate (CR/PR), *n* (%)	1 (3.0)	0	1 (3.0)

*Note*: Response criteria used was RECIST 1.1.

Abbreviations: CR, complete response; *N*, number of patients in intent‐to‐treat population; *n*, number of patients within category; PR, partial response; SD, stable disease.

**FIGURE 2:**
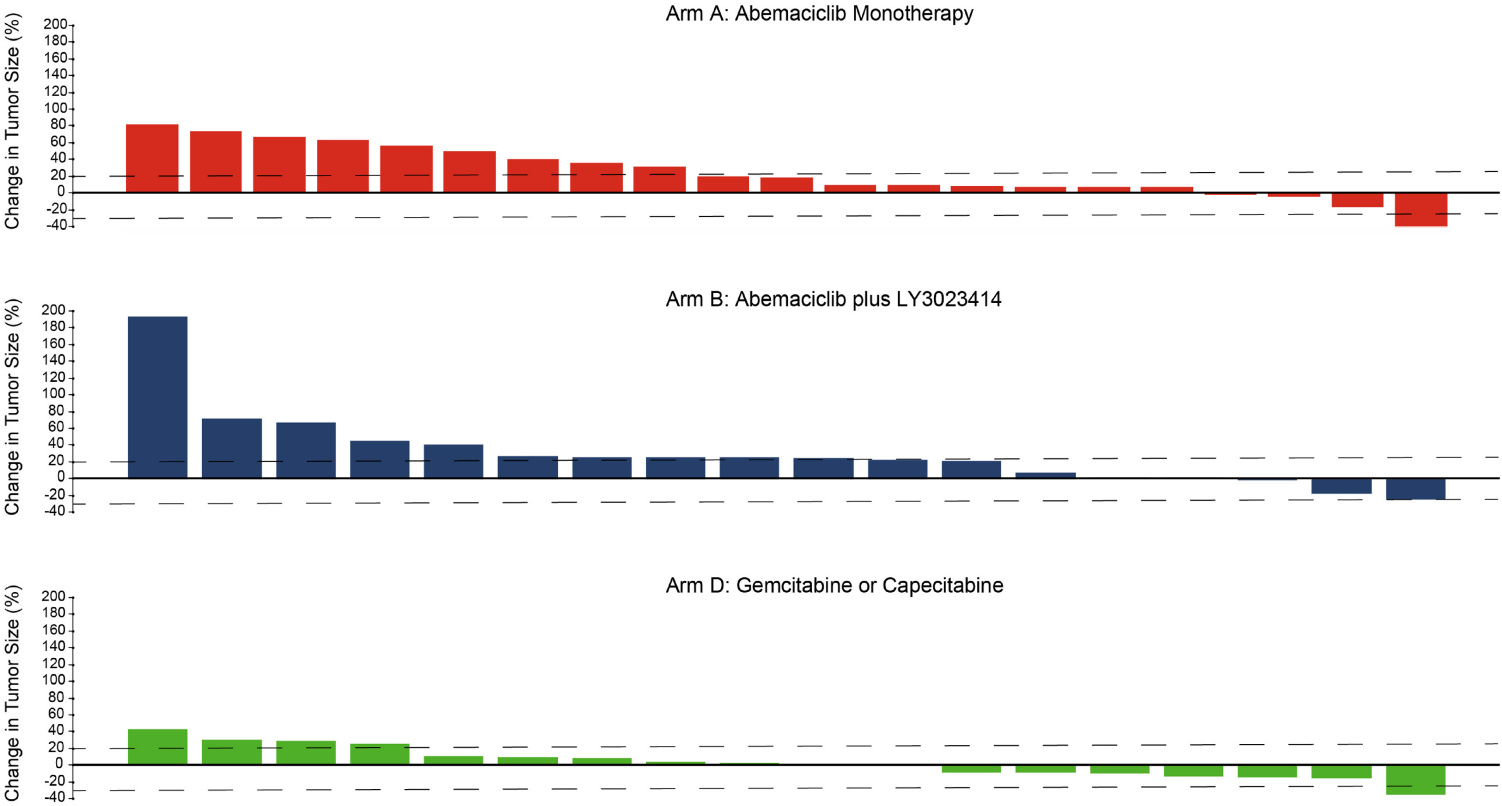
Best percentage change in tumor size in patients with measurable disease.

The best percentage change in tumor size relative to baseline was greater for those in Arm D than in any of the abemaciclib arms (Figure 2). One partial response was observed in Arms A and D, respectively. ORR was 3.0% (95% CI: 0.0–8.9) in Arm A, 0.0% (95% CI: N/A) for Arm B, and 3.0% (95% CI: 0.0–8.9) for Arm D (Table 2).

**FIGURE 3:**
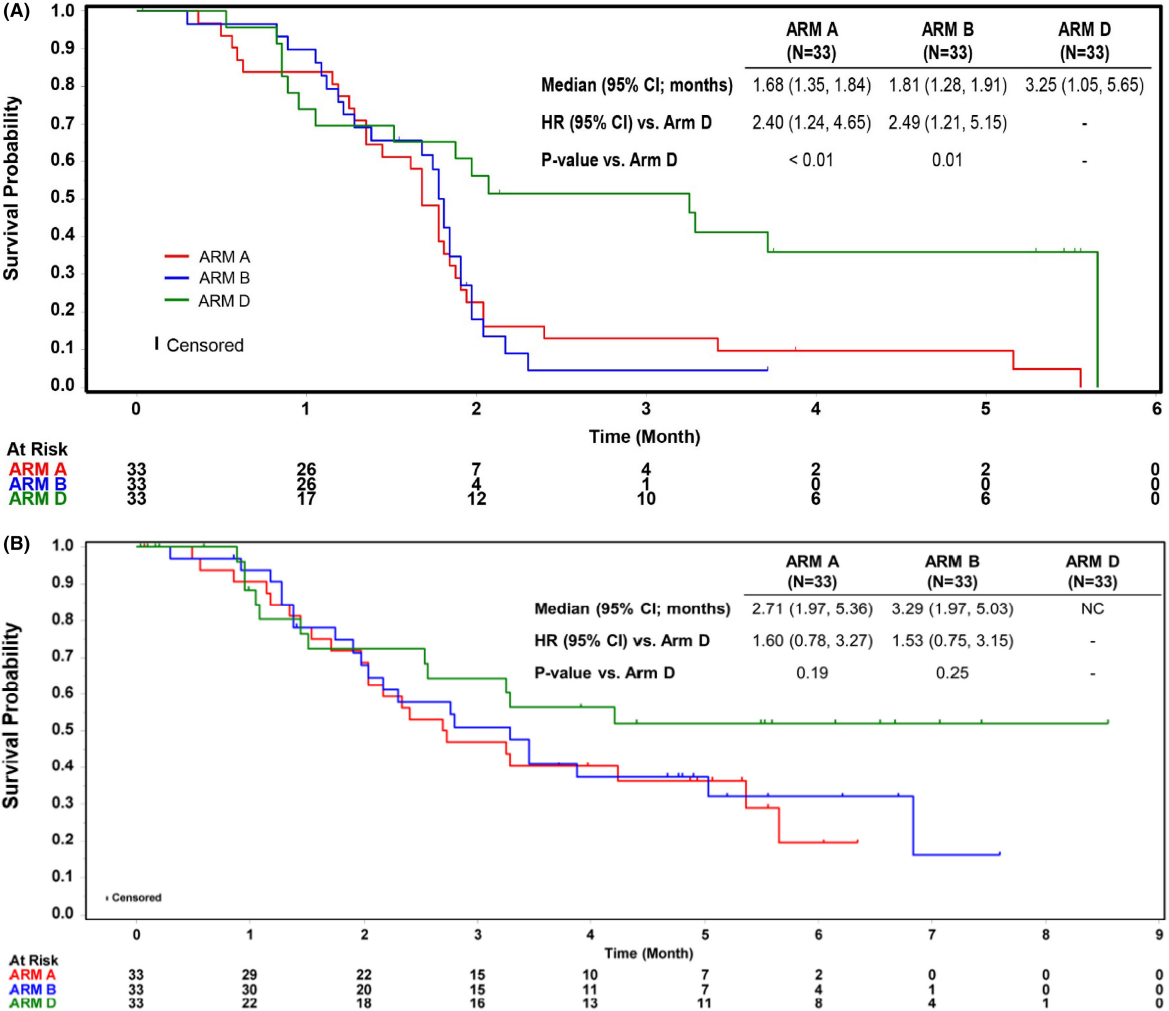
(A) Progression‐free survival in ITT population. (B) Overall survival in ITT population. ARM A, abemaciclib 200 mg; ARM B, abemaciclib 150 mg plus LY3023414; ARM D, SOC (gemcitabine or capecitabine); HR, Hazard Ratio; NC, not calculable. Hazard ratio was calculated using stratified Cox model with number of prior systemic therapy as the stratification factor. 2‐sided *p*‐value.

Median PFS was 1.7 months (95% CI: 1.35–1.84) for abemaciclib, 1.8 months (95% CI: 1.28–1.91) for abemaciclib plus LY3023414, and 3.3 months (95% CI: 1.05–5.65) for SOC (Figure 3A).

At the time of data cutoff, 22 deaths (66.7%) occurred in Arm A, 21 (63.6%) in Arm B, and 12 (36.4%) in Arm D. Median OS was 2.7 months (95% CI: 1.97–5.36) in Arm A and 3.3 months (95% CI: 1.97–5.03) in Arm B. Median OS was not reached in Arm D (Figure 3B). A stratified Cox model yielded a HR of 1.60 (95% CI: 0.78, 3.27) in Arm A and 1.53 (95% CI: 0.75, 3.15) in Arm B compared to SOC, indicating an unfavorable trend for the abemaciclib arms.

### 3.4 Safety

Treatment‐emergent adverse events (TEAEs) occurred in >99% of the treated patients (*n* = 98). The most commonly reported TEAEs, occurring in ≥15% of patients, are listed in Table 3. Grade 3 neutropenia and fatigue occurred in 1 patient each (14%) in the safety lead‐in. Gastrointestinal disorders were reported more frequently in Arm B compared to Arms A or D; no other marked differences were observed between arms.

**TABLE 3:** Treatment‐emergent adverse events occurring in ≥15% of patients (Safety Population).

	Safety lead‐in		Randomized stage 1					
	Abemaciclib + Galunisertib (*N* = 7), *n* (%)		Abemaciclib (*N* = 32), *n* (%)		Abemaciclib + LY3023414 (*N* = 33), *n* (%)		Gemcitabine or capecitabine (*N* = 26), *n* (%)	
	All grades	Grade 3/4	All grades	Grade 3/4	All grades	Grade 3/4	All grades	Grade 3/4
≥1 TEAE	7 (100.0)	5 (71.4)	31 (96.9)	25 (78.1)	33 (100.0)	20 (60.6)	26 (100.00)	21 (80.8)
Fatigue	5 (71.4)	2 (28.6)	19 (59.4)	5 (15.6)	17 (51.5)	6 (18.2)	12 (46.2)	3 (11.5)
Diarrhea	4 (57.1)	0 (0.0)	12 (37.5)	3 (9.4)	17 (51.5)	1 (3.0)	8 (30.8)	1 (3.8)
Nausea	5 (71.4)	0 (0.0)	9 (28.1)	0 (0.0)	16 (48.5)	2 (6.1)	10 (38.5)	2 (7.7)
Vomiting	2 (28.6)	0 (0.0)	10 (31.3)	2 (6.3)	16 (48.5)	1 (3.0)	9 (34.6)	4 (15.4)
Anemia	3 (42.9)	2 (28.6)	10 (31.3)	5 (15.6)	7 (21.2)	3 (9.1)	11 (42.3)	4 (15.4)
Thrombocytopenia	1 (14.3)	1 (14.3)	10 (31.3)	6 (18.8)^b^	11 (33.3)	7 (21.2)^c^	8 (30.8)	5 (19.2)^d^
Abdominal pain	4 (57.1)	0 (0.0)	9 (28.1)	2 (6.3)	6 (18.2)	1 (3.0)	9 (34.6)	1 (3.8)
Decreased appetite	4 (57.1)	0 (0.0)	9 (28.1)	1 (3.1)	9 (27.3)	1 (3.0)	8 (30.8)	1 (3.8)
Neutropenia	2 (28.6)	1 (14.3)^a^	8 (25.0)	8 (25.0)	2 (6.1)	2 (6.1)	4 (15.4)	4 (15.4)^d^
Stomatitis	0 (0.0)	0 (0.0)	0 (0.0)	0 (0.0)	13 (39.4)	5 (15.2)	7 (26.9)	1 (3.8)
Constipation	1 (14.3)	0 (0.0)	5 (15.6)	0 (0.0)	2 (6.1)	0 (0.0)	8 (30.8)	1 (3.8)

Abbreviations: *N*, number of patients in intent‐to‐treat population; *n*, number of patients within category; TEAE, treatment‐emergent adverse event.

^a^ Grade 4 neutropenia in 1 (14.3%) patient.

^b^ Grade 4 thrombocytopenia in 1 (3.1%) patient.

^c^ Grade 4 thrombocytopenia in 3 (9.1%) patients.

^d^ Grade 4 thrombocytopenia and neutropenia in 2 patients each [7.7%], respectively.

In Stage 1, 84.4%, 75.8%, and 88.5% of patients treated with abemaciclib, abemaciclib plus LY3023414, and SOC, respectively, experienced at least 1 Grade ≥3 TEAE, mostly hematologic toxicity and fatigue (Table 3).

Serious AEs regardless of causality were observed in 55.1% of patients. Two patients (28.6%) in the safety lead‐in, 4 (12.5%) in Arm A, 11 (33.3%) in Arm B, and 7 (26.9%) in Arm D experienced SAEs related to treatment. Nine patients (9.2%) died due to AEs while on treatment (*n* = 4; Arm B:3, Arm D:1) or within 30 days of discontinuation (*n* = 5; Arm A:2, Arm B:2, Arm D:1). Of these, 1 patient (Arm B) died due to tumor lysis syndrome, reported as possibly related to treatment. Dose adjustments due to AEs are summarized in Table S1.

### 3.5 Pharmacokinetics

The PK parameters for abemaciclib and galunisertib in the safety lead‐in arm and for abemaciclib plus LY3023414 in Arm B were consistent with the PKs observed in single agent studies (Table S2; Figure S2).

Similarly, steady‐state exposures for abemaciclib, its metabolites, galunisertib, and LY3023414 (Figure S3) were consistent with those observed in respective single‐agent studies.^28^, ^29^, ^30^, ^31^

## 4 DISCUSSION

*KRAS*‐mutated tumors, representing most pancreatic cancers, have overactive G1‐S cell cycle signaling, which drives cellular proliferation and tumor growth. Preclinical models demonstrated encouraging activity with CDK4/6 inhibitors, including synergism when dosed sequentially with taxane chemotherapy, by preventing DNA damage repair.^19^ This study aimed to improve DCRs and PFS with abemaciclib‐based therapies compared to SOC chemotherapy with gemcitabine or capecitabine. Given no data identifying cell cycle pathway aberrations predicting response to CDK4/6 inhibitors, our study enrolled an unselected patient population regarding CDK genomic alterations.

Following a safety lead‐in of abemaciclib plus galunisertib, testing of this combination was stopped by the sponsor for reasons unrelated to safety, and it did not advance to Stage 1. In Stage 1, no abemaciclib‐containing arms demonstrated DCRs superior to SOC chemotherapy in the ITT population (15.2% and 12.1% vs. 36.4%). Thus, no treatment arms advanced to Stage 2 and the study was closed early due to futility. Our primary endpoint was the DCR in the ITT population rather than the response‐evaluable population, due to our intent not to exclude patients who discontinued study treatment early due to clinical deterioration for either toxicities or cancer progression. In this study, 12 patients withdrew consent after starting treatment, some possibly due to clinical deterioration, 14 discontinued due to disease‐related adverse events or treatment‐related toxicities, and 7 patients died while on study treatment. This reflects an advanced cancer population with grim prognosis.

Median PFS was inferior with abemaciclib monotherapy (1.7 months) and abemaciclib in combination with LY3023414 (1.8 months) compared to standard chemotherapy (3.3 months). Of note, SOC performed better than anticipated (assumed median PFS of 1.5 months). OS rates were similarly lower in the abemaciclib‐containing arms compared with chemotherapy (abemaciclib: 2.7 months; abemaciclib plus LY3023414: 3.3 months; SOC: not reached). These results are comparable with those observed in a phase 1 study (*n* = 12) with the CDK4/6 inhibitor ribociclib plus mTOR inhibitor everolimus in patients with pretreated metastatic PDAC (PFS 1.8 months, OS 3.7 months).^37^ Additionally, palbociclib was studied in 12 patients with metastatic pancreatic cancer harboring CDKN2A genetic abnormalities and reported a PFS of 7.2 weeks and OS of 12.4 weeks.^38^ Altogether, these results are in line with those observed in the abemaciclib‐containing arms in our study.

To date, CDK4/6 inhibitors have been consistently ineffective for metastatic PDAC, possibly due to resistance mechanisms such as compensatory activation of the PI3K/mTOR and RAF/MAPK pathways.^37^ Several studies, including the one described herein, attempted to combine CDK4/6 blockade with PI3K/mTOR inhibition, but none have improved efficacy. More recently, preclinical and clinical reports suggested synergism between CDK4/6 and RAF/MAPK pathway inhibitors, including modulation of the tumor and immune microenvironment, and antitumor responses were observed in pancreatic cancer patients.^38^, ^39^, ^40^, ^41^

Hematologic side effects were common, but no significant differences were observed compared to SOC. More patients withdrew from treatment in Arm B compared to Arms A or D, possibly due to toxicity with increased rates of gastrointestinal AEs. Most common treatment‐related AEs with abemaciclib were fatigue, diarrhea, and nausea, whereas fatigue, anemia, and nausea were most common with SOC.

The PK profile of abemaciclib was consistent with previous evaluations in patients with advanced cancer,^28^ indicating no drug–drug interactions of clinical concern.

## 5 LIMITATIONS

Patients with metastatic PDAC have rapidly progressive disease, and few clinical trials, especially in the absence of a chemotherapy backbone, have demonstrated clinical benefit for patients with such advanced disease and an absence of targetable molecular alterations.^42^, ^43^ Our study was limited by enrolling a heavily pretreated population. Consequently, though not unexpected, a large number of patients discontinued study treatment early, likely due to clinical deterioration and adverse events, and having died of progressive disease before having a post‐baseline scan were non‐evaluable for response. The observed overlapping toxicities in the combination arm also contributed to early treatment discontinuations. Lastly, we were unable to perform biomarker studies due to the limited number of archival tumor samples; however, the lack of efficacy likely would have hindered any meaningful correlations.

## 6 CONCLUSION

Abemaciclib monotherapy or in combination with the PI3K/mTOR inhibitor LY3023414 did not improve DCR, PFS, or OS compared to standard chemotherapy in pretreated metastatic PDAC.

Given the aggressive nature of metastatic PDAC, molecularly guided interventions will need to account for complex signaling pathways and select biomarkers to identify patients most likely to benefit.^44^ While CDK4/6 inhibitors remain investigational in metastatic PDAC, the search for rational and tolerable therapeutic strategies that target resistance mechanisms continues.

## AUTHOR CONTRIBUTIONS

**E. Gabriela Chiorean:** Conceptualization (lead); data curation (equal); investigation (equal); resources (equal); supervision (equal); validation (equal); writing – original draft (equal); writing – review and editing (equal). **Vincent Picozzi:** Investigation (equal); resources (equal); writing – review and editing (equal). **Chung‐Pin Li:** Investigation (equal); resources (equal); writing – review and editing (equal). **Marc Peeters:** Investigation (equal); resources (equal); writing – review and editing (equal). **Joan Maurel:** Investigation (equal); resources (equal); writing – review and editing (equal). **Jaswinder Singh:** Investigation (equal); resources (equal); writing – review and editing (equal). **Talia Golan:** Investigation (equal); resources (equal); writing – review and editing (equal). **Jean‐Frédéric Blanc:** Investigation (equal); resources (equal); writing – review and editing (equal). **Sonya Chapman:** Conceptualization (equal); data curation (equal); formal analysis (equal); methodology (equal); project administration (equal); validation (equal); visualization (equal); writing – original draft (equal); writing – review and editing (equal). **Anwar M. Hossain:** Conceptualization (equal); data curation (equal); formal analysis (equal); methodology (equal); validation (equal); visualization (equal); writing – original draft (equal); writing – review and editing (equal). **Erica Johnston:** Conceptualization (equal); data curation (equal); project administration (equal); supervision (equal); validation (equal); writing – original draft (equal); writing – review and editing (equal). **Howard S. Hochster:** Investigation (equal); resources (equal); writing – review and editing (equal).

## FUNDING INFORMATION

The work was supported by Eli Lilly and Company (Indianapolis, IN, USA). Eli Lilly and Company had a role in the study design, collection, analysis, and interpretation of the data, writing of the manuscript, and submission of the manuscript for publication.

## CONFLICT OF INTEREST STATEMENT

E. Gabriela Chiorean reports personal fees from AstraZeneca, Bayer, Celgene, Eisai, Ipsen, Legend, Merck, Novartis, Noxxon, Pfizer, Seattle Genetics, Sobi, and Stemline, and grants from Boehringer–Ingelheim, Bristol–Myers Squibb, Celgene, Clovis, Corcept, Fibrogen, Halozyme, Incyte, Lonza, Lilly, MacroGenics, Merck, Rafael, Roche, and Stemline. V. Picozzi reports research funding from Abbvie, FibroGen, Ipsen, Merus, NGM Biopharmaceuticals, and Novocure and a consulting or advisory role with TriSalus Life Sciences, and stock and other ownership interests with Amgen and Johnson & Johnson. C‐P. Li reports no potential conflicts of interest with respect to the research, authorship, and/or publication of this article. M. Peeters reports no potential conflicts of interest with respect to the research, authorship, and/or publication of this article. J. Maurel reports research funding from Carlos III Health Institute; Catalan Agency for Management of University and Research Grants; Fundació la Marató de TV3; Olga Torres Foundation, and a consulting or advisory role with Advance Medical; Amgen; AstraZeneca; Bayer; Biocartis; Cancer Expert Now; Fundación Clínica Universitaria; Incyte; Merck; NanoString Technologies; Pierre Fabre; Roche; Sanofi; SERVIER; Shire; Sirtex Medical. J. Singh reports no potential conflicts of interest with respect to the research, authorship, and/or publication of this article. T. Golan reports honoraria from MSD, consulting or advisory roles with Abbvie, AstraZeneca, MSD, and Teva, speakers' bureau from Abbvie, AstraZeneca, and institute research funding from AstraZeneca and MSD. J‐F. Blanc reports consulting or advisory roles for Bayer, Ipsen, Eisai, Bristol–Myers Squibb, Roche and AstraZeneca, Servier, Incyte. S. C. Chapman, A. M. Hussain, and E. L. Johnston are full‐time employees of Eli Lilly and Company and are Eli Lilly and Company shareholders. H. Hochster reports personal fees from Bayer and Genentech and personal fees and nonfinancial support from Elion and TRIGR.

## ETHICS STATEMENT

The study was conducted in accordance with the 1964 Declaration of Helsinki and its later amendments, the International Conference on Harmonization Guidelines for Good Clinical Practice, and applicable local regulations. It was approved by the ethics committees of participating centers.

## PATIENT CONSENT STATEMENT

All patients provided written informed consent prior to participation in the study.

## Supporting information


Appendix S1

## Data Availability

Eli Lilly provides access to all individual participant data collected during the trial, after anonymization, with the exception of pharmacokinetic or genetic data. Data are available to request 6 months after the indication studied has been approved in the USA and EU and after primary publication acceptance, whichever is later. No expiration date for data requests is currently set once data are made available. Access is provided after a proposal has been approved by an independent review committee identified for this purpose and after receipt of a signed data sharing agreement. Data and documents, including the study protocol, statistical analysis plan, clinical study report, and blank or annotated case report forms, will be provided in a secure data sharing environment. For details on submitting a request, see the instructions provided at www.vivli.org.

## References

[1] Adamska A , Domenichini A , Falasca M . Pancreatic ductal adenocarcinoma: current and evolving therapies. Int J Mol Sci. 2017;18(7):1338. 28640192 10.3390/ijms18071338 PMC5535831

[2] Siegel RL , Miller KD , Fuchs HE , Jemal A . Cancer statistics, 2021. CA Cancer J Clin. 2021;71(1):7‐33. 33433946 10.3322/caac.21654

[3] Hosein AN , Brekken RA , Maitra A . Pancreatic cancer stroma: an update on therapeutic targeting strategies. Nat Rev Gastroenterol Hepatol. 2020;17(8):487‐505. 32393771 10.1038/s41575-020-0300-1 PMC8284850

[4] Von Hoff DD , Cridebring D , Tian OY , et al. Analysis of the role of plasma 25‐hydroxyvitamin D levels in survival outcomes in patients from the phase III MPACT trial of metastatic pancreatic cancer. Oncologist. 2021;26(4):e704‐e709. 33345430 10.1002/onco.13645 PMC8018329

[5] Conroy T , Ducreux M . Adjuvant treatment of pancreatic cancer. Curr Opin Oncol. 2019;31(4):346‐353. 30994497 10.1097/CCO.0000000000000546

[6] Ramanathan RK , McDonough SL , Philip PA , et al. Phase IB/II randomized study of FOLFIRINOX plus pegylated recombinant human hyaluronidase versus FOLFIRINOX alone in patients with metastatic pancreatic adenocarcinoma: SWOG S1313. J Clin Oncol. 2019;37(13):1062‐1069. 30817250 10.1200/JCO.18.01295 PMC6494359

[7] Van Cutsem E , Tempero MA , Sigal D , et al. Randomized phase III trial of pegvorhyaluronidase alfa with nab‐paclitaxel plus gemcitabine for patients with hyaluronan‐high metastatic pancreatic adenocarcinoma. J Clin Oncol. 2020;38(27):3185‐3194. 32706635 10.1200/JCO.20.00590 PMC7499614

[8] Wang‐Gillam A , Hubner RA , Siveke JT , et al. NAPOLI‐1 phase 3 study of liposomal irinotecan in metastatic pancreatic cancer: final overall survival analysis and characteristics of long‐term survivors. Eur J Cancer. 2019;108:78‐87. 30654298 10.1016/j.ejca.2018.12.007

[9] Huffman BM , Basu Mallick A , Horick NK , et al. Effect of a MUC5AC antibody (NPC‐1C) administered with second‐line gemcitabine and nab‐paclitaxel on the survival of patients with advanced pancreatic ductal adenocarcinoma: a randomized clinical trial. JAMA Netw Open. 2023;6(1):e2249720. 36602796 10.1001/jamanetworkopen.2022.49720 PMC9856813

[10] Noel MS , Kim S , Hartley ML , et al. A randomized phase II study of SM‐88 plus methoxsalen, phenytoin, and sirolimus in patients with metastatic pancreatic cancer treated in the second line and beyond. Cancer Med. 2022;11(22):4169‐4181. 35499204 10.1002/cam4.4768 PMC9678093

[11] Chun JW , Woo SM , Lee SH , et al. A real‐world analysis of nanoliposomal‐irinotecan with 5‐fluorouracil and folinic acid as third‐ or later‐line therapy in patients with metastatic pancreatic adenocarcinoma. Ther Adv Med Oncol. 2022;14:17588359221119539. 36062047 10.1177/17588359221119539 PMC9434681

[12] Mie T , Sasaki T , Takeda T , et al. Treatment outcomes of erlotinib plus gemcitabine as late‐line chemotherapy in unresectable pancreatic cancer. Jpn J Clin Oncol. 2021;51(9):1416‐1422. 34128055 10.1093/jjco/hyab091

[13] Hua J , Shi S , Liang D , et al. Current status and dilemma of second‐line treatment in advanced pancreatic cancer: is there a silver lining? Onco Targets Ther. 2018;11:4591‐4608. 30122951 10.2147/OTT.S166405 PMC6084072

[14] Vincent A , Herman J , Schulick R , Hruban RH , Goggins M . Pancreatic cancer. Lancet. 2011;378(9791):607‐620. 21620466 10.1016/S0140-6736(10)62307-0 PMC3062508

[15] Zeitouni D , Pylayeva‐Gupta Y , der C , Bryant K . KRAS mutant pancreatic cancer: no lone path to an effective treatment. Cancers (Basel). 2016;8(4):45. 27096871 10.3390/cancers8040045 PMC4846854

[16] Iriana S , Ahmed S , Gong J , Annamalai AA , Tuli R , Hendifar AE . Targeting mTOR in pancreatic ductal adenocarcinoma. Front Oncol. 2016;6:99. 27200288 10.3389/fonc.2016.00099 PMC4843105

[17] Jones S , Zhang X , Parsons DW , et al. Core signaling pathways in human pancreatic cancers revealed by global genomic analyses. Science. 2008;321(5897):1801‐1806. 18772397 10.1126/science.1164368 PMC2848990

[18] Franco J , Balaji U , Freinkman E , Witkiewicz AK , Knudsen ES . Metabolic reprogramming of pancreatic cancer mediated by CDK4/6 inhibition elicits unique vulnerabilities. Cell Rep. 2016;14(5):979‐990. 26804906 10.1016/j.celrep.2015.12.094 PMC4757440

[19] Salvador‐Barbero B , Álvarez‐Fernández M , Zapatero‐Solana E , et al. CDK4/6 inhibitors impair recovery from cytotoxic chemotherapy in pancreatic adenocarcinoma. Cancer Cell. 2020;37(3):340‐353. e6. 32109375 10.1016/j.ccell.2020.01.007

[20] Knudsen ES , Kumarasamy V , Ruiz A , et al. Cell cycle plasticity driven by MTOR signaling: integral resistance to CDK4/6 inhibition in patient‐derived models of pancreatic cancer. Oncogene. 2019;38(18):3355‐3370. 30696953 10.1038/s41388-018-0650-0 PMC6499706

[21] Liu F , Korc M . Cdk4/6 inhibition induces epithelial‐mesenchymal transition and enhances invasiveness in pancreatic cancer cells. Mol Cancer Ther. 2012;11(10):2138‐2148. 22869556 10.1158/1535-7163.MCT-12-0562 PMC3752412

[22] Franco J , Witkiewicz AK , Knudsen ES . CDK4/6 inhibitors have potent activity in combination with pathway selective therapeutic agents in models of pancreatic cancer. Oncotarget. 2014;5(15):6512‐6525. 25156567 10.18632/oncotarget.2270 PMC4171647

[23] Dickler MN , Tolaney SM , Rugo HS , et al. MONARCH 1, a phase II study of abemaciclib, a CDK4 and CDK6 inhibitor, as a single agent, in patients with refractory HR(+)/HER2(−) metastatic breast cancer. Clin Cancer Res. 2017;23(17):5218‐5224. 28533223 10.1158/1078-0432.CCR-17-0754 PMC5581697

[24] Sledge GW Jr , Toi M , Neven P , et al. MONARCH 2: abemaciclib in combination with fulvestrant in women with HR+/HER2‐ advanced breast cancer who had progressed while receiving endocrine therapy. J Clin Oncol. 2017;35(25):2875‐2884. 28580882 10.1200/JCO.2017.73.7585

[25] Johnston S , Martin M , di Leo A , et al. MONARCH 3 final PFS: a randomized study of abemaciclib as initial therapy for advanced breast cancer. NPJ Breast Cancer. 2019;5:5. 30675515 10.1038/s41523-018-0097-z PMC6336880

[26] Royce M , Osgood C , Mulkey F , et al. FDA approval summary: abemaciclib with endocrine therapy for high‐risk early breast cancer. J Clin Oncol. 2022;40(11):1155‐1162. 35084948 10.1200/JCO.21.02742 PMC8987222

[27] U.S. Food & Drug Administration . FDA expands early breast cancer indication for abemaciclib with endocrine therapy. 2023 https://www.fda.gov/drugs/resources‐information‐approved‐drugs/fda‐expands‐early‐breast‐cancer‐indication‐abemaciclib‐endocrine‐therapy

[28] Patnaik A , Rosen LS , Tolaney SM , et al. Efficacy and safety of abemaciclib, an inhibitor of CDK4 and CDK6, for patients with breast cancer, non‐small cell lung cancer, and other solid tumors. Cancer Discov. 2016;6(7):740‐753. 27217383 10.1158/2159-8290.CD-16-0095

[29] Kim ES , Kelly K , Paz‐Ares LG , et al. Abemaciclib in combination with single‐agent options in patients with stage IV non–small cell lung cancer: a phase Ib study. Clin Cancer Res. 2018;24(22):5543‐5551. 30082474 10.1158/1078-0432.CCR-18-0651

[30] Bendell JC , Varghese AM , Hyman DM , et al. A first‐in‐human phase 1 study of LY3023414, an oral PI3K/mTOR dual inhibitor, in patients with advanced cancer. Clin Cancer Res. 2018;24(14):3253‐3262. 29636360 10.1158/1078-0432.CCR-17-3421

[31] Rodon J , Carducci MA , Sepulveda‐Sánchez JM , et al. First‐in‐human dose study of the novel transforming growth factor‐beta receptor I kinase inhibitor LY2157299 monohydrate in patients with advanced cancer and glioma. Clin Cancer Res. 2015;21(3):553‐560. 25424852 10.1158/1078-0432.CCR-14-1380 PMC4337847

[32] Smith TJ , Bohlke K , Lyman GH , et al. Recommendations for the use of WBC growth factors: American Society of Clinical Oncology clinical practice guideline update. J Clin Oncol. 2015;33(28):3199‐3212. 26169616 10.1200/JCO.2015.62.3488

[33] Rizzo JD , Somerfield MR , Hagerty KL , et al. Use of epoetin and darbepoetin in patients with cancer: 2007 American Society of Clinical Oncology/American Society of Hematology clinical practice guideline update. J Clin Oncol. 2008;26(1):132‐149. 17954713 10.1200/JCO.2007.14.3396

[34] NCI . Common Terminology Criteria for Adverse Events (CTCAE) Version 4.0. 2009 https://evs.nci.nih.gov/ftp1/CTCAE/CTCAE_4.03/Archive/CTCAE_4.0_2009‐05‐29_QuickReference_8.5x11.pdf

[35] Eisenhauer EA , Therasse P , Bogaerts J , et al. New response evaluation criteria in solid tumours: revised RECIST guideline (version 1.1). Eur J Cancer. 2009;45(2):228‐247. 19097774 10.1016/j.ejca.2008.10.026

[36] Kaplan EL , Meier P . Nonparametric estimation from incomplete observations. J Am Stat Assoc. 1958;53(282):457‐481.

[37] Weinberg BA , Wang H , Witkiewicz AK , et al. A phase I study of Ribociclib plus everolimus in patients with metastatic pancreatic adenocarcinoma refractory to chemotherapy. J Pancreat Cancer. 2020;6(1):45‐54. 32642630 10.1089/pancan.2020.0005 PMC7337242

[38] Baghdadi TA , Halabi S , Garrett‐Mayer E , et al. Palbociclib in patients with pancreatic and biliary cancer with CDKN2A alterations: results from the targeted agent and profiling utilization registry study. JCO Precis Oncol. 2019;3:1‐8. 10.1200/PO.19.00124 35100714

[39] Kato S , Adashek JJ , Shaya J , et al. Concomitant MEK and cyclin gene alterations: implications for response to targeted therapeutics. Clin Cancer Res. 2021;27:2792‐2797. 33472910 10.1158/1078-0432.CCR-20-3761 PMC11005753

[40] Knudsen ES , Kumarasamy V , Chung S , et al. Targeting dual signalling pathways in concert with immune checkpoints for the treatment of pancreatic cancer. Gut. 2021;70(1):127‐138. 32424005 10.1136/gutjnl-2020-321000 PMC7671951

[41] Maust JD , Frankowski‐McGregor CL , Bankhead A III , Simeone DM , Sebolt‐Leopold JS . Cyclooxygenase‐2 influences response to cotargeting of MEK and CDK4/6 in a subpopulation of pancreatic cancers. Mol Cancer Ther. 2018;17(12):2495‐2506. 30254182 10.1158/1535-7163.MCT-18-0082 PMC6279520

[42] Ho WJ , Jaffee EM , Zheng L . The tumour microenvironment in pancreatic cancer ‐ clinical challenges and opportunities. Nat Rev Clin Oncol. 2020;17(9):527‐540. 32398706 10.1038/s41571-020-0363-5 PMC7442729

[43] Singh RR , O'Reilly EM . New treatment strategies for metastatic pancreatic ductal adenocarcinoma. Drugs. 2020;80(7):647‐669. 32306207 10.1007/s40265-020-01304-0 PMC7466866

[44] Schoninger SF , Blain SW . The ongoing search for biomarkers of CDK4/6 inhibitor responsiveness in breast cancer. Mol Cancer Ther. 2020;19(1):3‐12. 31909732 10.1158/1535-7163.MCT-19-0253 PMC6951437

