# Supplementary material for: Efficacy and safety of abemaciclib alone and with PI3K/mTOR inhibitor LY3023414 or galunisertib versus chemotherapy in previously treated metastatic pancreatic adenocarcinoma: A randomized controlled trial
Source: Cancer Med. 2023 Oct 16;12(20):20353–64. doi: 10.1002/cam4.6621 (PMC10652308; doi:10.1002/cam4.6621)

**Supplemental Table 1. Toxicity Management - Summary of Dose Adjustments from Adverse Events (Safety Population)**

|  | **Safety Lead-in**  **(N=7)** | | **Arm A**  **(N=32)** | **Arm B**  **(N=33)** | | **Arm D**  **(N=26)** |
| --- | --- | --- | --- | --- | --- | --- |
|  | **Abemaciclib** | **Galunisertib** | **Abemaciclib** | **Abemaciclib** | **LY3023414** | **Gemcitabine or Capecitabine** |
| Patients with Dose Reduction; n (%) | 3 (42.9) | 1 (14.3) | 10 (31.3) | 10 (30.3) | 8 (24.2) | 12 (46.2) |
| Patients with Dose Omission; n (%) | 4 (57.1) | 3 (42.9) | 24 (75.0) | 22 (66.7) | 22 (66.7) | 10 (38.5) |
| Discontinuation due to AE; n (%) | 0 (0.0) | | 4 (12.5) | 8 (24.2) | | 6 (23.1) |

## **Supplemental Table 2. Pharmacokinetic Parameter Estimates for Abemaciclib, Total Analytes (Abemaciclib+M2+M20), and Galunisertib During the Safety Lead-In**

|  | **Geometric Mean (CV%)** | | | | | | |
| --- | --- | --- | --- | --- | --- | --- | --- |
|  | **Abemaciclib** | | **Total Analytes^a^** | | | **Galunisertib** | |
| **Parameter** | **Cycle 1 Day 1^b^**  **(N=6)** | **Steady State^c^**  **(N=4)** |  | **Cycle 1**  **Day 1^b^**  **(N=6)** | **Steady State^c^**  **(N=4)** | **Cycle 1 Day 1^b^**  **(N=6)** | **Steady State^c^**  **(N=4)** |
| C_max_ (ng/mL) | 66.4 (67) | 356 (137) |  | 183^a^ (79) | 1280^a^ (82) | 704 (65) | 1140 (43) |
| t_max_^d^ (h) | 6.50 (4.05-7.00) | 4.01 (1.00-6.00) |  | 6.50 (4.05-7.00) | 2.50 (0.00-6.00)^e^ | 1.1 (1.0-4.0) | 1.5 (0.5-4.0) |
| AUC(0-6h) (ng·h/mL) | 192 (98) | 1620 (121) |  | 527^a^ (122) | 5920^a^ (73) | 2360 (72) | 3580 (44) |

Abbreviations: AUC(0-6h), area under the concentration time curve from zero to 6 hours; C_max_ = maximum observed plasma concentration; CV, coefficient of variation; h, hour; n, number of observations; t_max_, time of maximum observed drug concentration.

^a^ Total analytes = sum of abemaciclib and its 2 metabolites, M2 and M20. Values quoted are in nM for Cmax and nM*h for AUC parameters.

^b^ Single-dose PK parameter calculations excluded Subject 1012 due to missing times

^c^ Multiple-dose PK parameters calculated on Day 13 (n=1), Day 14 (n=2), or Day 15 (n=1). Subject 1004 excluded from analysis due to dose reduction, Subject 1002 excluded due to missing times and Subject 1012 excluded due to being outside sampling window.

^d^ Median (range).

^e^ t_max_ for Subject 1011 reported at time zero. This could possibly be due to PK predose samples being drawn after dosing instead of before. Subject was included in the summary statistics

## **Supplemental Figure 1. I3Y-MC-JPCJ Study Design**


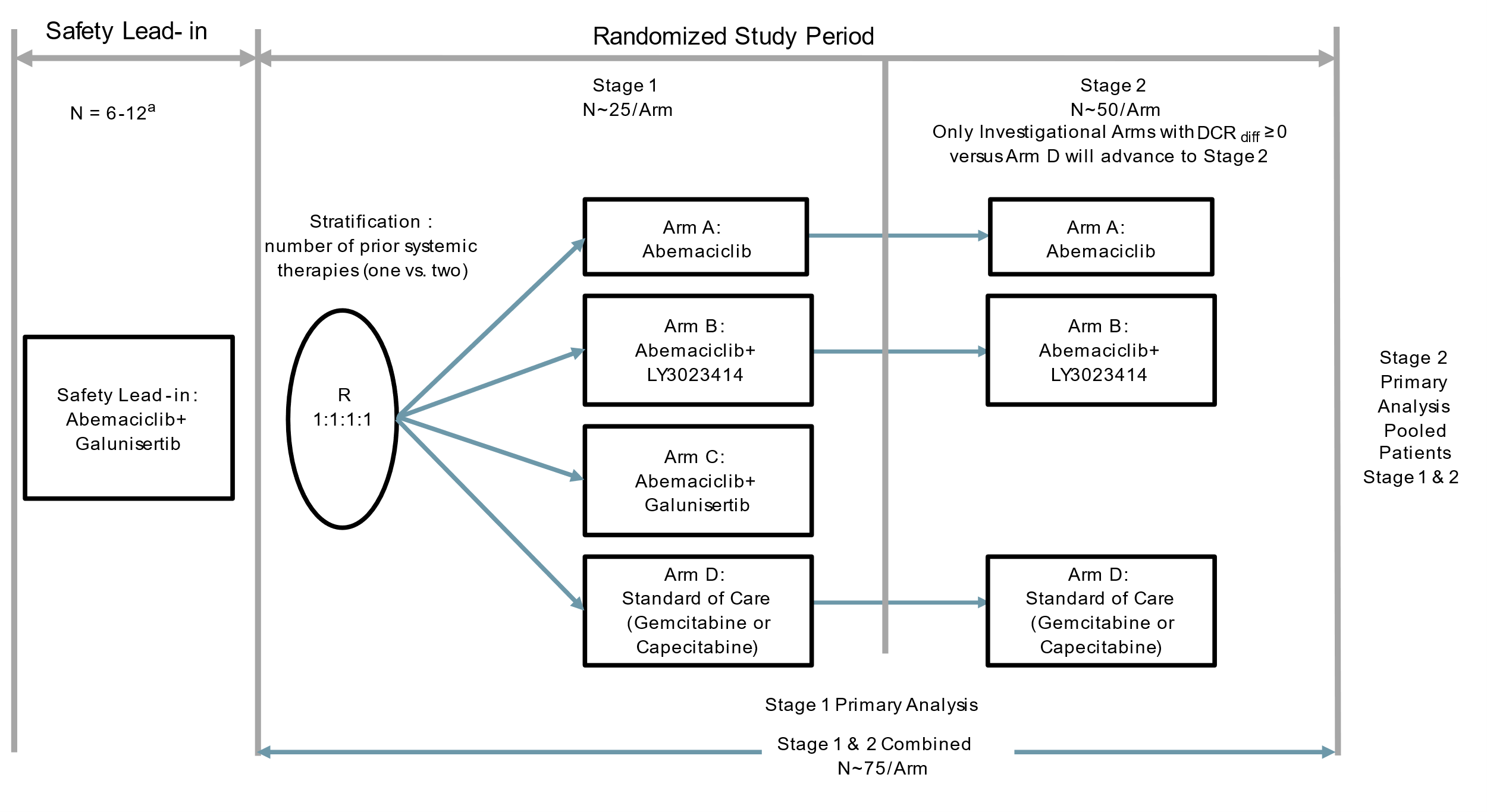


Abbreviations: ECOG = Eastern Cooperative Oncology Group; DCR diff = disease control rate difference; N = number of patients; R = randomize; RECIST v1.1 = Response Evaluation Criteria in Solid Tumors Version 1.1; PS = performance status.

^a^ Safety lead-in estimate.

## **Supplemental Figure 2. Mean plasma concentration-time curves for abemaciclib (left), total active analytes (abemaciclib+M2+M20) (center), and galunisertib (right) during the safety lead-in on Cycle 1 Day 1 after a single dose (red) or on Day 14 after twice daily dosing (blue).**

Abbreviations: SD = standard deviation.

## **Supplemental Figure 3. Individual plasma concentrations of abemaciclib (left) when abemaciclib was given alone (Arm A) or in combination with LY3023414 (Arm B) or galunisertib (Safety Lead-In), and concentrations of LY3023414 when given in combination with abemaciclib (Arm B) (right).**


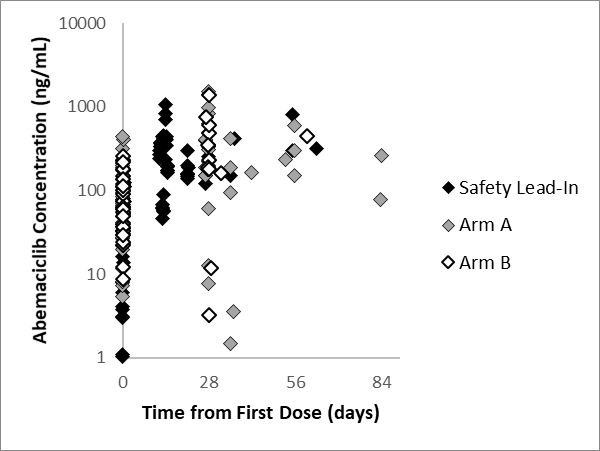

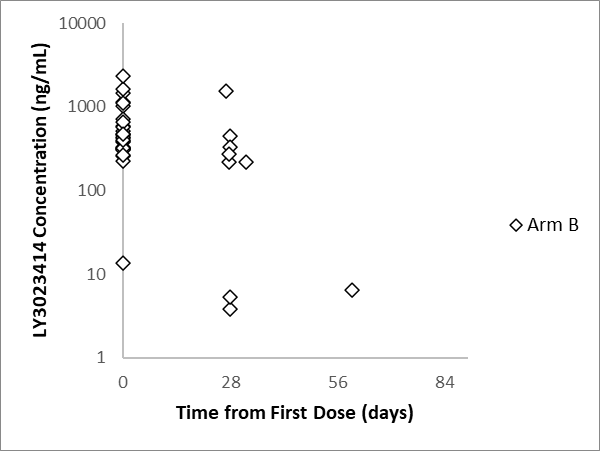

Supplement: Supplementary file 1 — Appendix S1 [file CAM4-12-20353-s001.docx]
